# The Effect of pH on Rabbit Septal Cartilage Shape Change: Exploring the Mechanism of Electromechanical Tissue Reshaping

**Published:** 2014-06-27

**Authors:** Lauren E. Tracy, Brian J. Wong

**Affiliations:** Department of Otolaryngology—Head and Neck Surgery, University of California, Irvine, Calif

**Keywords:** cartilage, cartilage reshaping, electromechanical reshaping, EMR, mechanism

## Abstract

**Introduction:** Electromechanical reshaping (EMR) involves the application of an electrical current to mechanically deformed cartilage to create sustained tissue shape change. Although EMR may evolve to become an inexpensive and reliable way of producing shape change in cartilage during reconstructive surgery, the precise mechanism of EMR is unknown. We aim to examine the isolated effect of protonation (pH) on shape change in cartilage. **Methods:** Nasal septal cartilages of rabbits were mechanically deformed and placed in a rigid jig. The deformed cartilages were submerged in isotonic phosphate buffered saline baths (osm = 290 mmol/Kg) with a pH of 3 (N = 51), pH of 7 (N = 51), and a pH of 11 (N = 51) for 15 minutes. Following re-equilibration, specimens were removed from their jig and the angle change from baseline was measured using digital micrometry. **Results:** Significant shape change was noted in all specimens, with an angle change of 33.6°, 33.3°, and 32.0° experienced by the pH of 3, 7, and 11 groups, respectively. Despite a trend toward increased shape change in the acidic treatment, there was no significant difference between groups. **Conclusions:** Although current evidence indicates that dynamic oxidation-reduction reactions within the extracellular matrix of cartilage may be implicated in EMR-induced shape change, when pH was isolated as a single variable it was not sufficient to produce cartilage shape change.

Cartilage is a tissue of paramount importance for the structural frameworks of the face and the functionality of the upper airway. The nuances of human expression and sensation rely in part upon shape and structure established by the cartilage scaffolds of the nose and external ear. Likewise, the patency of the human airway depends on support from cartilage in the nasal airway, trachea, and larynx. Over recent years, electromechanical reshaping (EMR) has emerged as an experimental method to reshape cartilage and other tissues.^1^^-^5 EMR involves the application of a direct electric current to electrodes contacting mechanically deformed cartilage within regions of increased internal stress, with subsequent stress relaxation achieved as a consequence of *in situ* redox reactions. The potential medical applications of EMR are broad and include esthetic procedures such as rhinoplasty and otoplasty, as well as procedures aimed at restoring normal function such as septoplasty, reconstruction of facial structures, and correction of congenital or acquired malformations of the airway. Although EMR may evolve to become an inexpensive and reliable way of producing shape change in cartilage, the mechanisms responsible for EMR-induced shape change remain unclear.

Extensive and detailed *ex vivo* studies have found correlation between the degree of cartilage shape change during EMR and the total charge transferred, voltage difference, and current application time.^1^^-^7 In vivo studies in animal models have demonstrated potential clinical efficacy.^8^ The complex, osmotically active, and negatively charged extracellular matrix of cartilage behaves like a charged hydropolymer and is susceptible to redox reactions and alteration of ionic bonds, van der Waals interactions, and hydrogen bonding. In EMR, an electrical potential is established resulting in (1) the electrolysis of water (near anode) and reduction of water (near cathode); (2) creation of chemical gradients throughout the extracellular matrix; and (3) the evolution of hydrogen, oxygen, and chlorine gasses.^2^ Electrolysis of water has the potential to alter macromolecular structure within the matrix including status of hydrogen bonds, charge shielding, and van der Waals interactions. In particular, we suspect that EMR induces protonation of electronegative proteoglycans in the extracellular matrix, thereby affecting hydrogen bonding and local electrochemical repulsive forces in cartilage resulting in changes in the Donnan equilibrium, at least transiently.^1^^,^^9^ This study aimed to isolate and evaluate the effect of steady-state pH changes alone, without the complex alterations created by electrolysis, on cartilage shape change.

## METHODS

### Tissue specimens

**Figure 1:**
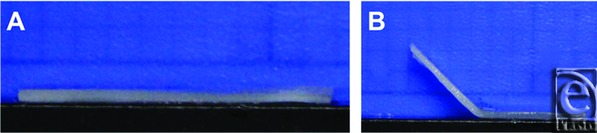
Rabbit septal cartilage. (*a*) Rabbit septal cartilage in its native unbent state, viewed on its short axis. (*b*) The same specimen after being reshaped in the 90° jig in a treatment pH solution.

Nasal septal cartilages (N = 153) were extracted from freshly euthanized New Zealand white rabbits (age 9-12 weeks) procured from a local abattoir (Fig 1). The dimensions (size and shape) of these specimens vary little between individual animals when controlled for animal age or weight.^10^ Each septum was cut into 24 ± 0.5 mm × 6 ± 0.2 mm sections and placed in a phosphate buffered saline (PBS) solution for immediate use.

### pH solutions

Three different solutions were created using PBS titrated to a pH of 3, 7, and 11, respectively, with 10M hydrochloric acid and 10M sodium hydroxide (Sigma-Aldrich, St Louis, Missouri). The solutions were buffered using minimal NaCl (Sigma-Aldrich, St Louis, Missouri) to a physiological osmolality of 290 ± 4, and osmolality was confirmed using a vapor pressure osmometer (Wescor, Logan, Utah).

### Experimental set-up

**Figure 2:**
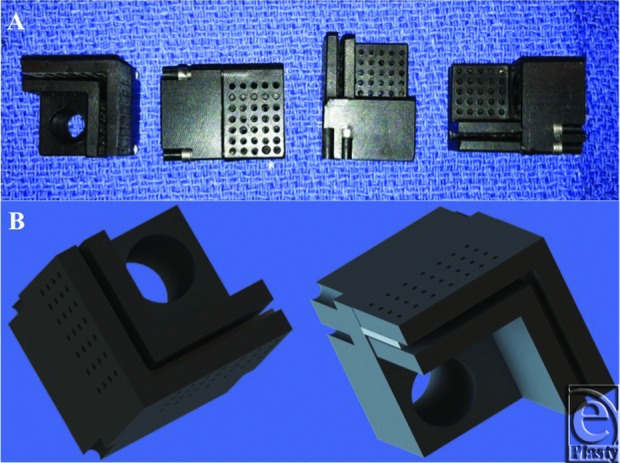
Plastic jig used to mechanically deform specimens during pH treatment. (*a*) The 90° jig with multiple perforations to allow free flow of liquid to the cartilage specimen. (*b*) The jig was designed to hold the cartilage specimen statically in position without tension or pressure on the specimen.

**Figure 3:**
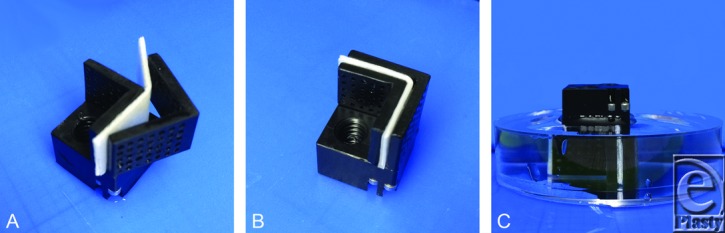
Experimental set-up. (*a*) Cartilage specimens are placed in the perforated hard plastic jig. (*b*) Using set screws to gently fasten the jig walls, the cartilage is mechanically deformed to 90°. (*c*) Following mechanical deformation, the jigged cartilage specimen is completely submerged in the pH treatment bath.

**Figure 4:**
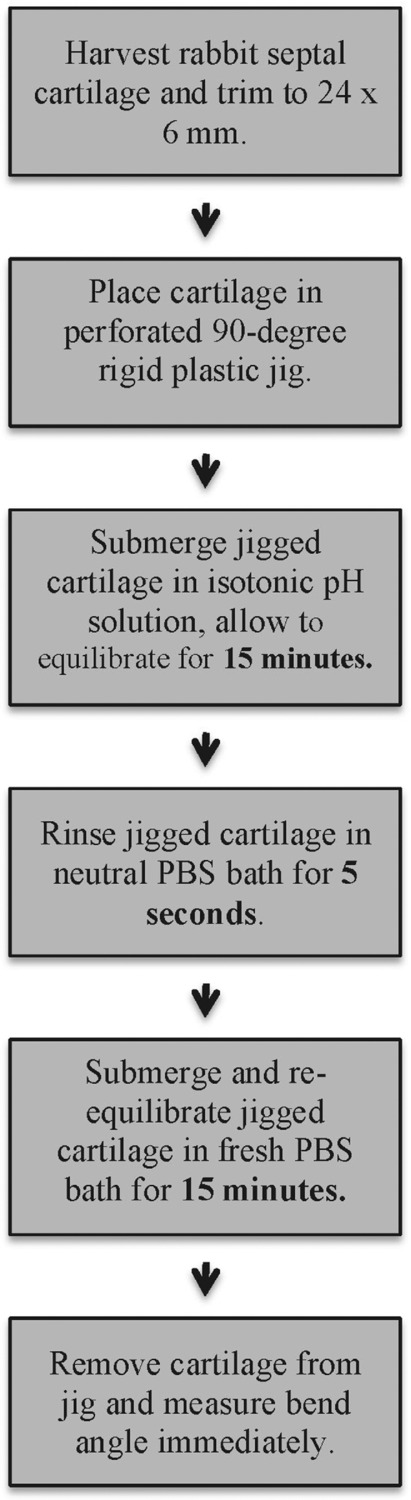
Experimental flowchart.

Rigid plastic jigs were created to hold the cartilage specimen at a 90° bend angle. Multiple perforations were created in the jig walls to increase contact between the cartilage and the surrounding aqueous media (Fig 2). After placement in the plastic jig, each cartilage specimen was secured with set screws in these 90° jigs, and then submerged completely in a bath with pH of 3 (N = 51), pH of 7 (N = 51), or pH of 11 (N = 51) for 15 minutes (Fig 3). The jigged specimen was stirred and tapped to ensure the release of any air bubbles present on the cartilage-liquid interface. Following the pH bath immersion, the jigged cartilages were washed in PBS with neutral pH of 7 briefly and then allowed to re-equilibrate by submersion in a fresh PBS bath with pH of 7 for 15 minutes (all the while remaining mechanically deformed in the jig) (Fig 4). After this final PBS bath, the cartilage jig was patted dry with a surgical towel, and the cartilage specimen was immediately removed from the jig and digitally photographed on its long axis (Digimax i5, Samsung, Ridgefield Park, New Jersey).

### Analysis of bend angles

The bend angle of the treated specimens was determined using the protractor function available on ImageJ (National Institute of Health, Rockville, Maryland). Native rabbit septal cartilage is uniformly flat with a bend angle of 0°, whereas the theoretical maximum bend angle achievable with our jig is 90° (Fig 1). A smaller outside angle correlates with less effective reshaping of the cartilage. Statistical analysis was performed using a Kolmogorov-Smirnov test.

## RESULTS

**Figure 5:**
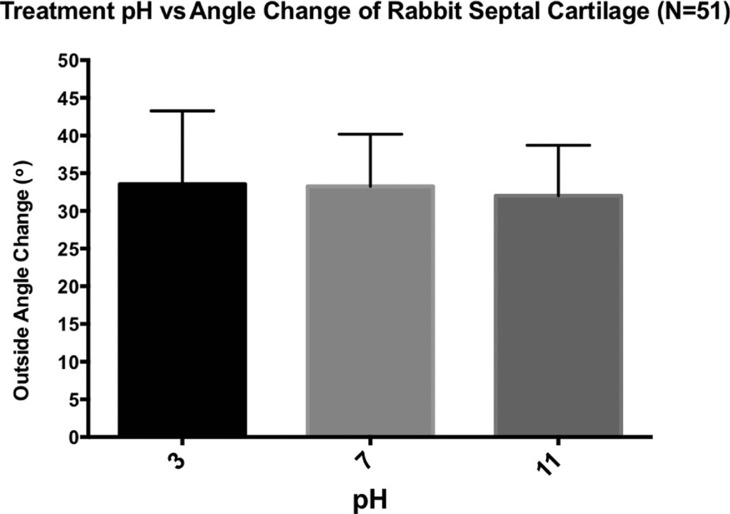
Effect of pH on cartilage shape change. Cartilage specimens were immersed for a total of 15 minutes in an isotonic pH treatment bath, then allowed to re-equilibrate for 15 minutes in neutral pH PBS solution before undergoing digital photography and analysis of their bend angle change. The specimens treated with pH of 3, 7, and 11 experienced angle changes of 33.6°, 33.3°, and 32.0°, respectively. There was no significant difference between any groups (n = 51, *P* > .3). Error bars represent SD. PBS indicates phosphate buffered saline.

All cartilage specimens experienced shape change, with an angle change of 33.6°, 33.3°, and 32.0° experienced by the pH of 3, 7, and 11 groups, respectively (N = 51/group) (Fig 5). Despite a trend toward increased shape change (as reflected by greater outside angle change) in the acidic treatment, there was no significant difference between the pH of 7 control group and the experimental pH of 3 group (*P* = .33), or between the pH of 7 control group the experimental pH of 11 group (*P* = .52). Likewise, there was no significant difference between the pH of 3 and pH of 11 groups (*P* = .45).

## DISCUSSION

The extracellular matrix of cartilage is composed of 65% to 80% water, 15% to 25% collagen, and 3% to 10% proteoglycans.^11^ The proteoglycans in collagen exist as polyanionic molecules with numerous affiliated carboxyl and sulfate groups having pK_a_ values ranging from 3 to 3.6.^12^ The most prevalent proteoglycan in cartilage, aggrecan, has more than a hundred negatively charged chondroitin sulfate side chains, with each chain in turn have some 50 carboxy and sulfate groups each.^13^ The highly negatively charged and immobile proteoglycans present in cartilage exert a high Donnan osmotic swelling pressure in the cartilage that is balanced and mitigated by the mechanical tensile strength of collagen within the tissue.^9^ This complex system of dynamic negative charge in a rigid molecular framework allows cartilage tissue to behave largely like a matrix of immobilized charge bathed in a highly conductive fluid. Prior experimentation using streaming potential and isometric stress has shown that the isoelectric point of intact cartilage is approximately at pH of 2.75, and that this isoelectric curve was dominated by the titration of carboxyl groups.^12^ The isoelectric point of cartilage (pH = 2.75) as well as the pK_a_ values of cartilage associated carboxyl and sulfate groups (pH = 3–3.6) suggest that cartilage approaches its maximal electroneutrality around pH 3. For this reason, we felt that maximal cartilage shape change would be induced following exposure to pH 3 treatment baths. Treatment with pH 7 represented a physiologic control, and treatment with pH 11 represented an extreme basic control of equal magnitude to the acidic pH 3 group. Given the known pK_a_s of cartilage proteoglycan groups, it is probable that treatment with solutions of pH less than 3 would result in increasing polybasic protonation of cartilage macromolecules, which would intensify molecular repulsions and made shape change less likely.

Previous studies have shown that during EMR, total charge transfer correlates with the degree of cartilage shape change and that electrolysis of water clearly occurs and is the dominant chemical reaction. Hydrolysis will drop the local pH at the anode and raise the pH at the cathode. Hence, investigating whether pH alone might be a dominant mechanism responsible for EMR-induced cartilage shape change is important. The most straightforward approach to examine the effect of steady state pH change is through the use of immersion baths; however, the stable and widespread changes in pH created by immersion are very different from that which occurs during EMR in situ.

This is the first attempt to examine pH changes alone as a causative factor responsible for macroscopic cartilage shape change in EMR, decoupled from redox chemistry and evolved electrical and chemical gradients. In this first attempt, we observed no significant difference in shape change between acidic (pH 3), neutral (pH 7), and basic pH (pH 11).

While our results indicate that modification of steady state pH alone is not sufficient to produce cartilage shape change, it is important to note that pH alterations during EMR are much more dynamic (both spatially and temporally) than the experimental model used in this study. EMR produces unique electrical and chemical gradients around the anode and cathode. As ions are transported along these gradients, they are able to interact with various matrix constituents^1^^,^^2^ These transient gradients and the local creation and diffusion of charged species is likely relevant to the shape change effect observed with EMR. When a cartilage specimen is flexed, compression occurs at the inner bend angle while tension occurs along the outer bend angle. Hence, the local density of charged proteoglycans is increased along the inner bend angle of the cartilage specimen as matrix molecules are brought into closer contact. Conversely, as the cartilage matrix is decompressed along the outer bend angle of the cartilage specimen, the local charge density is decreased. By precise positioning of the anode and cathode, EMR is able to dramatically and rapidly increase the rate of redox reactions in areas of maximal proteoglycan charge density, while minimally affecting redox reactions elsewhere in the cartilage sample (unpublished data). In comparison to EMR, immersion rapidly creates steady state changes in pH throughout the specimen without localizing changes in species concentration to a specific area of cartilage in particular regions of increased internal stress. Notably, due to the continuous and discrete application of current, the milieu immediately adjacent to the anode or cathode may have a significantly different pH from the surrounding tissue and the immersion media.

In addition to hydrolysis causing changes in pH, EMR is likely to produce a variety of redox reactions with the resultant generation of other chemical species. In addition to hydrogen gas, we also know that chlorine gas is generated during treatment of cartilage with EMR. Although the specific molecular reactions propagated by EMR are not known at this time, it is an active area of investigation in our laboratory and those of others. It is highly likely that the mechanism of EMR is not dependent upon any steady state change in one variable (such as pH), but on the variety of temporally and spatially localized changes incurred by application of a directed electrical current.

Importantly, our previous published works have shown that much less dramatic shape change is observed in cartilage control groups when using jigs and specimen sizes similar to those used in this study. Typically, control group rabbit septal cartilages (to which electrical current has not been applied) demonstrated bend angles in the range of 10° to 19°.^1^^,^^2^^,^^4^^,^^8^ There were some differences. First, those control specimens were submerged in an isotonic aqueous media for only 4 to 20 minutes total, which is less than the total 30-minute submersion used in this study. In addition, the plastic jigs used in previous experiments did not have perforations, but had solid walls and a base in direct contact with the cartilage specimen, thus reducing diffusion of aqueous media to only the exposed top and sides of the jig. This jig setup, in addition to the reduced submersion time used in previous studies, could have prevented complete diffusion of the surrounding aqueous media throughout the cartilage specimens, thus reducing the effect of biomechanical changes (swelling, osmotic pressure gradients, hydrolysis) known to result from prolonged osmotic stress. Finally, specimens are secured within the jig using multiple small setscrews. It is possible that in this study, there was a systematic use of a greater compression force when tightening these setscrews that could create localized fractures at the flexure point.

There are a number of limitations to our study. The mechanical 90° bend angle imposed on the natively straight septal cartilage by the hard plastic jig may lead to trauma or microfracture in the specimen. Although rabbit cartilage is pliable, and studies on cartilage tissue treated similarly with a 90° angle jig have demonstrated good ex vivo chondrocyte viability, and even long-term in vivo survival, it is possible that the large number of specimens used in this experiment incurred varying amounts of mechanical trauma from setscrew tightening.^2^^,^^8^ Even if not grossly visible, any disruption of the cartilage's structural framework would certainly alter the microenvironment of the cartilage extracellular matrix in a way that would impair or obscure a modest experimental effect, while not necessarily affecting chondrocyte viability. To reduce the possibility of microtrauma to the cartilage specimen, in future experiments, we plan to use a hard plastic jig with a more obtuse bend angle. Similarly, future experiments will include histologic analysis of specimens to exclude the role of microtrauma in cartilage reshaping. Although experience with prior studies and the lack of significant shape change induced with the current model make microtrauma an unlikely player in our results, histologic evaluation may still yield useful mechanistic information.

This study represents a first attempt at analyzing the effect of varying pH on cartilage shape change. While no significant shape change was associated with acidic or basic treatments in the current study, this experiment brings to light the dynamic and spatially complex electrochemical gradients induced by EMR. Hopefully, the results of this study can be used to guide and improve future work in elucidating the electrochemical mechanism of EMR.

## CONCLUSIONS

Although prior work suggests that redox reactions within the extracellular matrix of cartilage is the likely mechanism of EMR-induced cartilage shape change, this study shows that steady state changes in pH alone may not be sufficient to induce shape change to a degree greater than a pH neutral control. Thus, an isolated change in pH within the cartilage environment is not responsible for the functionality of EMR. Likely, the mechanism of EMR is more complex than simple pH change and probably involves multiple dynamic and localized reactions. Further studies are needed to elucidate the precise mechanism of EMR cartilage reshaping, with thought to a more encompassing and dynamic assessment of molecular changes in the cartilage extracellular environment.
